# Maximizing the Information Content of Ill-Posed Space-Based Measurements Using Deterministic Inverse Method

**DOI:** 10.3390/rs10070994

**Published:** 2018

**Authors:** Prabhat K. Koner, Prasanjit Dash

**Affiliations:** 1 Earth System Science Interdisciplinary Center, University of Maryland, 5825 University Research Ct., College Park, MD 20740, USA; 2 NOAA/NESDIS Center for Satellite Applications and Research, E/RA3, 5830 University Research Ct., College Park, MD 20740, USA; 3 Global Science and Technology, Inc., and affiliate CIRA, Colorado State University, Fort Collins, CO 80523, USA

**Keywords:** ozone profile retrieval, deterministic inverse, regularized total least square, Tropospheric Emission Spectrometer (TES), Cross-track Infrared Sounder (CrIS), surface temperature, optimal estimation method (OEM)

## Abstract

For several decades, operational retrievals from spaceborne hyperspectral infrared sounders have been dominated by stochastic approaches where many ambiguities are pervasive. One major drawback of such methods is their reliance on treating error as definitive information to the retrieval scheme. To overcome this drawback and obtain consistently unambiguous retrievals, we applied another approach from the class of deterministic inverse methods, namely regularized total least squares (RTLS). As a case study, simultaneous simulated retrieval of ozone (O_3_) profile and surface temperature (ST) for two different instruments, Cross-track Infrared Sounder (CrIS) and Tropospheric Emission Spectrometer (TES), are considered. To gain further confidence in our approach for real-world situations, a set of ozonesonde profile data are also used in this study. The role of simulation-based comparative assessment of algorithms before application on remotely sensed measurements is pivotal. Under identical simulation settings, RTLS results are compared to those of stochastic optimal estimation method (OEM), a very popular method for hyperspectral retrievals despite its aforementioned fundamental drawback. Different tweaking of error covariances for improving the OEM results, used commonly in operations, are also investigated under a simulated environment. Although this work is an extension of our previous work for H_2_O profile retrievals, several new concepts are introduced in this study: (a) the information content analysis using sub-space analysis to understand ill-posed inversion in depth; (b) comparison of different sensors for same gas profile retrieval under identical conditions; (c) extended capability for simultaneous retrievals using two classes of variables; (d) additional stabilizer of Laplacian second derivative operator; and (e) the representation of results using a new metric called “information gain”. Our findings highlight issues with OEM, such as loss of information as compared to a priori knowledge after using measurements. On the other hand, RTLS can produce “information gain” of ~40–50% deterministically from the same set of measurements.

## 1. Introduction

Ozone (O_3_) is the third most important gas that enhances greenhouse effect. In the stratosphere, O_3_ prevents ultraviolet radiation from negatively affecting life on Earth’s surface. At the same time, O_3_ in the troposphere is considered to be a pollutant e.g., [1]. From a modelers’ perspective O_3_ is one of the most important input parameters for understanding of the fundamental atmospheric processes. It is a prerequisite for many climate and atmospheric studies and is labelled as an Essential Climate Variable (ECV) by the Global Climate Observation System (GCOS). Although satellite-based ozone retrievals from UV-VIS measurements are popular, the nadir-viewing infrared (IR) measurement is particularly advantageous for nighttime retrievals. The first satellite instrument designed to measure the vertical distribution of ozone was the backscatter ultraviolet (BUV) spectrometer instrument on NIMBUS 4 launched in 1970. Since 1970, many publications e.g., [1–9] have been published on atmospheric O_3_ estimation from several satellite-based instruments (e.g., CrIS, MIPAS, OMI, MLS, GOME, TOMS, OMPS, SBUV/2, SCIAMACHY, ACE-FTS, IASI, AIRS and TES, see Appendix A for additional acronyms). Unfortunately most of these retrievals are made using stochastic methods, where errors are treated as information. In this study, we focus on nadir-viewing thermal IR (TIR) instruments for O_3_ profile retrieval using deterministic inverse. With nadir-view, retrieval of ozone profiles is relatively easier than those of other trace gases since the mixing ratio of O_3_ increases with altitude unlike most other trace gases. This reduces the complexity of the Hilbert function as well as the condition number of the inverted matrix. Also, plenty of O_3_ absorption lines with variable line strengths at 9.7 μm increases the information content of the retrieval.

To investigate and compare the uncertainty in the satellite retrieval problem, a simulated comparative study with a deterministic method and a prevalent stochastic method for profile retrievals from satellite hyper-spectral IR measurements is undertaken. The definition of deterministic and stochastic methods can be found in [10–13]. This work is extended from our previous studies [13–16] by including more case studies to convince the scientific community about the flaws of prevailing strong reliance on Bayesian probability-based methods employed by major space and environmental agencies. The basic inverse method will remain similar to that described in our earlier publication [13,16], but different fundamental aspects of ill-posed inversion will be discussed including more case studies using both deterministic and stochastic methods.

Among the existing deterministic methods e.g., [17–21] the family of regularized total least squares (RTLS) methods has a well-established heritage in other branches of science, especially medical imaging e.g., [22–26]. However, it has seldom been exploited in Earth observation science to date. We selected the RTLS that is the only one which has a mathematical form to determine the optimal regularization strength using all noise terms embedded in the residual vector e.g., [13] and monotonically reduces the regularization strength towards a solution point. Other data-driven deterministic methods, i.e., generalized cross-validation (GCV) and L-Curve, are unstable to dynamically calculate the regularization at all iterations. A recent study shows that these methods are unsuitable for satellite inverse problems [18], based on pre-calculated regularization strength using GCV and L-Curve.

Our implementation of RTLS employing a Laplacian first derivative operator (LFDO) as a stabilizer injects added information into the inversion. The LFDO constrains the solution since the update of adjacent atmospheric parameters in a profile are close, which is less harmful than the use of an a priori of what are significantly dynamic atmospheric parameters. Theoretically, the final RTLS solution is independent of the initial guess parameters of targeted retrievals and regularization is data-driven at all iterations. A successful implementation of a similar algorithm (termed as “Modified Total Least Squares” or MTLS) in the near-real time (NRT) operational environment [27,28] for sea surface temperature (SST) retrievals has been in effect since August 2013 for three operational geostationary satellite sensors (GOES-13, GOES-14 and GOES15) at the Office of Satellite and Product Operations (OSPO), National Oceanic and Atmospheric Administration (NOAA). This is a relatively low ill-conditioned problem (typical condition number of Jacobian ~5) and is solved by a single iteration. The paradigm shift in operational inverse method is providing NRT high-quality SST data to the community with a 50% reduction in error, as compared to the previous stochastic (regression) method. The implementation has been made after careful investigation of comparative results from various inverse methods. It is worth mentioning that OEM error is always higher than least squares (LS) error see Figure 11 [28] for such a simple problem, which is expected because additional error input into the retrieval system increases the error in OEM output.

Similar algorithms employing MTLS or truncated total least squares (TTLS) on offline SST retrieval using MODIS-A L1B data [29,30] have significantly improved the retrieval results. This provides us with strong confidence that the proposed physically deterministic method can also be successfully employed in real-time operations for hyper-spectral measurements. We have published preliminary simulated results [13,14], where it was found that satellite hyperspectral retrieval problems can be uniquely solved using the RTLS method for simulated retrievals (IR and microwave). We demonstrated successful O_3_ retrieval using RTLS [16] for a limited observation, where balloon-based occultation FTIR measurements were used with very high spectral resolution (0.004 cm^−1^). The horizontal spatial resolution of nadir-viewing atmospheric measurement is high compared to limb/occultation, which makes it attractive to understand local as well as global atmospheric processes. This study will focus on the simultaneous simulated retrieval of O_3_ and ST from the nadir-viewing satellite-based FTIR measurements of CrIS, Cross-Track Infrared sounder, with a spectral resolution of 0.625 cm^−1^) and TES, Tropospheric Emission Spectrometer, with a spectral resolution 0.06 cm^−1^. These results have also been compared against a prevalent method using OEM. We have made significant progress in this field, but we realize that more is needed to convince the scientific community of our findings, and to secure support from space agencies to make further progress through wider implementation of deterministic techniques in satellite retrieval problems.

The key points of the retrieval algorithm using RTLS method for remote sensing measurement are rewritten for the convenience of readers in Section 2. Simultaneous smooth profile of O_3_ and surface temperature using RTLS for two different infrared sounders CrIS and TES will be presented in Section 3. The information content using sub-space analysis for understanding of the noise propagation from measurement to the state space under ill-conditioned matrix will be discussed in Section 3.3. Section 4 includes the simulated retrievals from realistic ozonesonde data. The retrievals from selective profiles of ozonesonde data is explained in Sections 4.1 and 4.2 for two different methods of RTLS and OEM, respectively. Sections 4.3 and 4.4 include the comparative retrievals between methods of RTLS and OEM for both CrIS and TES. The comparative error analysis in terms of “information gain” between methods and sensors will be discussed in Section 4.5. Conclusion is made in Section 5.

## 2. Methodology

The theoretical foundation of an IR remote sensing forward model is Schwarzschild’s equation of radiative transfer (RT). In a non-scattering atmosphere under local thermodynamic equilibrium, the following equation governs the transfer of emitted TIR radiance (I_λ_ (t)) at nadir that reach the top of the atmosphere (TOA) cf. [31] at a given wavenumber, λ:

(1) $${\text{I}}_{\text{λ}}(\text{t})={\text{I}}_{\text{λ}}\left({\text{z}}_{0}\right)\text{τ}\left({\text{z}}_{0},\text{z}\right)+\int_{{\text{z}}_{0}}^{\text{z}}{\text{J}}_{\text{λ}}\left({\text{z}}^{\prime }\right)\left(\frac{\text{dτ}\left({\text{z}}^{\prime },\text{z}\right)}{{\text{dz}}^{\prime }}\right){\text{dz}}^{\prime }$$

where, z_0_ and z are atmospheric height at the surface and TOA respectively; and z′ is the in between two, J_λ_ is the emission from a particular layer and τ is transmissivity, I_λ_ (z_0_) represents the radiation at the surface and τ(z_0_,z) is total transmissivities between the surface and TOA.

A Fourier transform spectroscopic instrument typically works in the IR region and measures the radiance at a finite number of spectral points with equidistant wavenumbers. Therefore, a suitable discretization process is used over the integrals in Equation (1). There are many different discretization possibilities: simple classic quadrature method, collocation points and nodes, degenerate kernel approximations (by Eigen functions or by orthonormal systems or approximation by Taylor series or interpolation) and projection methods (Galerkin moment or least squares). We have employed GENSPECT [32] for our forward modeling, which is a line-by-line (LBL) RT model (RTM) that uses a degenerate kernel function. The discretization process of RT equations leads to a set of nonlinear system of equations, which are in a Hilbert space. To solve the above-mentioned Hilbert function, the quadratic constrained RTLS problem stated in [13,16,33–37] is used as follows:

(2) $$\frac{min}{\Delta x\in X}\phi (x,y)\frac{\|K\Delta x-\Delta y{\|}^{2}}{1+\|\Delta x{\|}^{2}}\;\;subject\;to\;\|L\Delta x{\|}^{2}\leq \delta^{2}$$

the term ***L*** denotes the regularization operator, *ϕ*(**x, y**) is the cost function and δ is infinitesimal. ***K*** is the Jacobian, Δ**x** is the update of the state space at the *i*th iteration and Δ**y** is the residual. The detailed derivation of applied RTLS for current problem is available in [13,16] and the final form is

(3) $$\Delta x={\left(K^{T}K-g(x)I+\alpha^{2}L^{T}L\right)}^{-1}K^{T}\Delta y_{\delta }$$

where, $\text{g}(x)=\frac{{\left\|y_{\delta }-K\Delta x\right\|}^{2}}{1+\|\Delta x{\|}^{2}},\alpha$ is regularization strength, Δ*y*_δ_ is the residual including measurement noise and *I* is the identity matrix. Although fully explained in [13], the working mechanism of RTLS method is reiterated in this paragraph for the sake of completeness and for the convenience of the readers. For the calculation of g(*x*), an update of ***x*** is required, which is obtained as Δ*x* = 0 for the first iteration and the retrieved Δ*x* for successive iterations. Even though considering Δ*x* = 0 for the first iteration, the second regularization term of RTLS stabilizes the solution by the value of ***α***, which is calculated using the same value of Δ*x*. Thus, any under- or over-estimate of Δ*x* is compensated by the value of ***α*** and it is stated as a dual-regularized method. The success of any regularization method is dependent on the correct regularization strength for every iteration and characteristics of the regularization operator. Conventionally Laplacian first derivative operator (LFDO) is used as a stabilizer (***L*** in Equation (2)), in the case of a profile retrieval problem, it provides additional information and is a better approximation compared to regularization using the identity matrix [15]. Using a LFDO, this is done by forcing values of the update of adjacent points within a profile to be close. Moreover, it is difficult to develop a mathematical derivation for a nonlinear problem (i.e., RT equation) and RTLS derivation is also based on linear problem. Thus, the ***I*** matrix is empirically replaced by the ***L*** matrix in Equation (3) to compensate the effect of nonlinearity. The final form of RTLS at the *i*th iteration as:

(4) $$x_{i+1}=x_{i}+{\left(K^{T}K-g(x)L^{T}L+\alpha^{2}L^{T}L\right)}^{-1}K^{T}\Delta y_{\delta }$$

It is worth mentioning that the RTLS does not belong to either stochastic or Tikhonov or empirical regularization methods. It is derived from the understanding of quadratic eigenvalue analysis of matrix inversion, which is equivalent to the minimization of the Rayleigh-Quotient equation [33]. For each measurement instance, the optimal regularization strength (***α***) is calculated at all iterations to block the nonlinear error injection into the retrieved space, as well as to restrict propagation of other errors as described earlier for all measurement instances. The regularization strength (***α***) of the RTLS method is data driven, and is calculated from the residual vector as:

(5) $$W=L^{-T}\left(K^{T}K-g(x)L\right)L^{-1}$$

The lowest singular value of the matrix *W* has been shown to provide the optimal regularization strength [33]. Since the RTLS method is a dimensionless formulation and keeps uniformity of two separate classes of variables of the present problem, we consider a logarithmic scale for both state space parameters and measurements to produce a unit-less Jacobian without changing the functional property of the problem, i.e.,

(6) $$K=\frac{\partial (\log y)}{\partial (\log x)}=\frac{x}{y}\frac{\partial y}{\partial x}$$

## 3. Simulated Theoretical Smooth Profile Retrieval

Readers may be wonder, why simulated data are used rather than real data. This is because radiative transfer equations are complex functions that cannot be easily approximated by an explicit class of function (e.g., quadratic, convex, logarithmic). Thus, it is difficult to prove theoretically only by mathematical derivation that one algorithm is a better choice over the other for profile retrievals from RT-based real remote sensing measurements where associated errors cannot be exactly characterized. The sources of such errors are enormous, such as instrument error, forward model error, spectral error, line shape error (line overlapping, far wing effect of major molecules, line mixing etc.), errors from minor interfering gases or unmodeled parameters, background RT error and nonlinearity error cf., [16]. To avoid these hurdles, comparative numerical experiments constraining the problem close to realistic situations considering original inverse methods are the best choice. We urge that simulation-based assessment is one of the most critical steps but is often under-appreciated. This provides us with exact “ground truth” to analyze performances. Also, with simulations, inputs can be controlled to exactly what is needed for a particular purpose and allow us to either exclude or include regulated operational problems (calibration, fast forward model error, cloud detection). This allows us to concentrate on the performances of the inversion methods only. Moreover, it will be better to evaluate the performances of stochastic methods under the correct error covariances for the simulated set, which is not possible in the retrievals from real measurement because error is not quantifiable in global measurement instances.

### 3.1. Profile Retrievals from Simulated CrIS Measuremts

The sensor specifications of the CrIS hyper-spectral sounder onboard Suomi National Polar-orbiting Partnership (NPP) is considered in this study since this instrument will continue to be flown in a series of Joint Polar Satellite System (JPSS) missions until at least 2038. For real data from CrIS, channel radiance is given by a convolution of the instrument line shape (ILS) function with the monochromatic radiance from the simulation at the entrance to the interferometer [38]. For the simulated retrieval study, a simplified “sinc” function is considered to produce equivalent CrIS measurements by convolving with the simulated spectrum. For example, the simulated measurement is calculated by the spectra for O_3_ at a resolution of 0.06 cm^−1^ using the GENSPECT LBL model, for the US 1976 standard atmospheric temperature and O_3_ profiles, and ST of 300 K and surface emissivity of one and convolved with the “sinc” function. To achieve a more realistic condition, we applied our “select channel” algorithm [16] to discard the channels, which are contaminated by the radiance from other interfering trace gases (e.g., CO_2_, N_2_O, CH_4_, SO_2_, NO_2_, NH_3_, HNO_3_, OCS, HOCl, H_2_O_2_, H_2_O and H_2_S). For example, “select channel” algorithm congregate 169 channels out of 302 channels for a selected window (900–1090 cm^−1^). Monte Carlo noise realizations were added to calculated spectrum of 1% (signal to noise ratio, SNR = 100), which is conservative because reported CrIS SNR is more than 150 for longwave IR (LWIR) channels e.g., [39,40], to produce equivalent realistic measurement. During retrieval, we also consider the surface temperature (ST) as an additional retrieved parameter and the first guess is arbitrarily set to 275 K.

**Figure 1.**
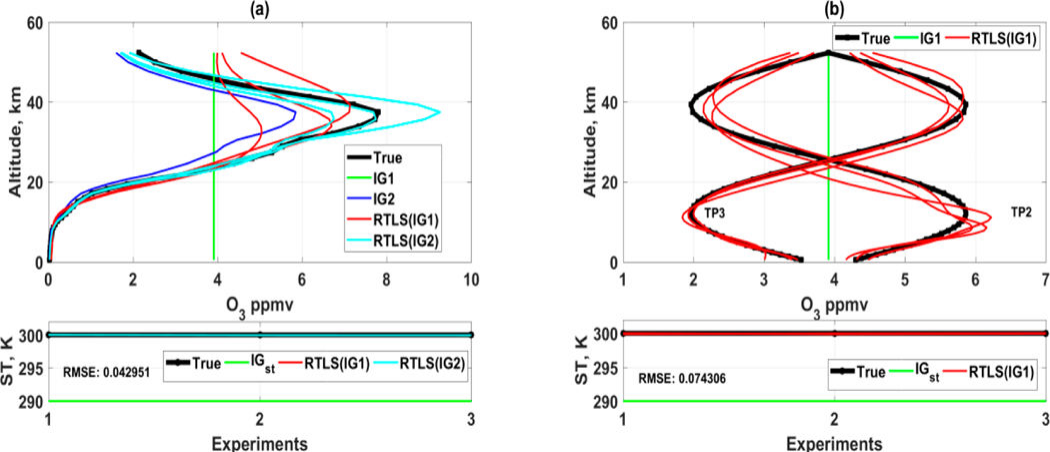
Simultaneous retrievals of O_3_ and surface temperature using RTLS from simulated CrIS measurements; Upper-panel: O_3_ profiles; Lower-panel: surface temperature: (**a**) retrieved O_3_ profiles (solid red from IG1 and cyan from IG2) from two different IGs (IG1 is green and IG2 is blue) for realistic true profile (solid black) and (**b**) retrieved O_3_ profiles (solid red) from IG1 (solid green) only for two different sinusoidal true profiles (solid black).

Retrievals adding Monte Carlo noise in the simulated spectrum have been made using RTLS for three different true profiles (TP), which are TP1 (realistic), and TP2 and TP3 (extreme case full-sinusoidal profiles). TP1 is a 1976 US standard O_3_ profile for the earth’s atmosphere. To improve confidence on the outcome, two initial guess (IG) profiles, one is a constant (IG1, green) and the other is a realistic (0.8 times of TP1, IG2, blue), are considered as shown in Figure 1. Only TP1 is solved from IG2 (Figure 1a). We have purposefully done this simulated experiment using two unrealistic sinusoidal profiles to understand the inverse properties of RT function and it does not violate any limits from the point of RT physics. It is obvious that parameters can go beyond the boundary for a specific iteration when Newtonian iterative optimization is used in a nonlinear problem. Also, the solution of sinusoidal profiles will give us an additional advantage to understand and analyze altitudinal information content for such measurements.

The simulated retrieval result confirms as shown in Figure 1a that CrIS measurement can retrieve good O_3_ realistic profiles up to ~30 km using RTLS method without any a priori information from two different IGs (IG1 and IG2) for TP1. However, a higher deviation in solution is observed for TP2 at ~10 km and at same time the oscillation for TP3 at ~10 km is lower as compared to that of TP2. This implies that there is a weak space for such measurement at 10~15 km and the severity of weak space depends on the shape of the profile (see Figure 1b). The profile retrievals of TP1 above 30 km are noisy even when solved from IG2, which confirms that the information available from CrIS measurement is low. On the contrary, tire profile retrieval of TP2 is improved above 30 km and some oscillation is observed for TP3 at that level. “This implies that the information of retrieval is not only dependent on CrIS measurements but also significantly dependent on the shape of the profiles resulting in different information contents. The retrieval of surface temperature is extremely good, where the root mean square errors of 6 retrievals of both sets are 0.04 and 0.07 K for realistic and sinusoidal protiles resppctively.

### 3.2. Profile Retrievals from Simulated TES Measuremts

Profile retrievals using also simulated Tropospheric Emission Spectrometer measurements onboard the; Aura spacecraft following the same approach as for CrIS are conducted in this study. The “select channel” algorithm leaves us 956 out of 3167 channels for the same selected window (900–1090 cm^−1^), which can be used for an O_3_ retrieval without using any interfering gases in the forward model. The number of selected channels is ~5.6 times higher than that of CrIS measurement because the TES-instrument is designed with ~10 times higher spectral resolution (0.06 cm^−1^) than CrIS (0.625 cm^−1^). Effective TES measurements are generated by adding random noise at the rate of SNR = 300 on top) of the simulated spectrum as specified by the noise level of TES.

**Figure 2.**
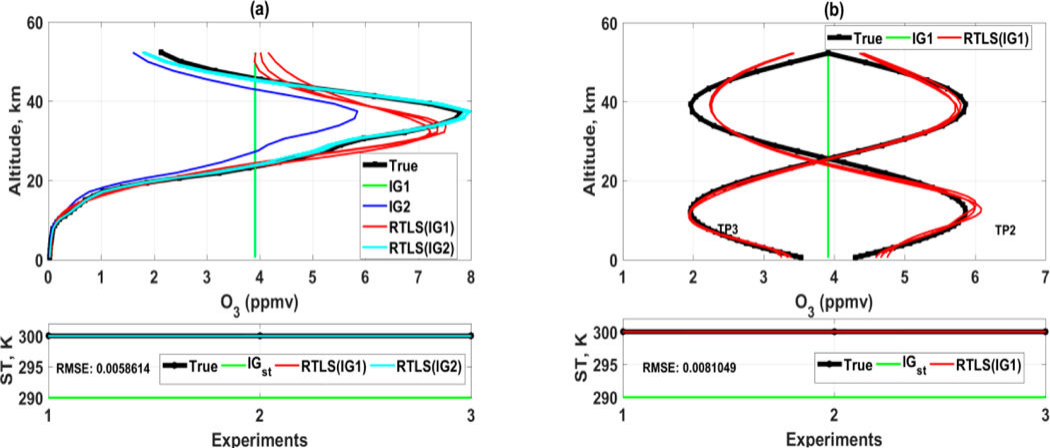
Similar to Figure 1 but for TES retrievals.

As observed in Figures 1 and 2, the retrievals of O_3_ profiles and surface temperature from simulated TES spectrum are improved compared to those of CrIS. This is expected because of both higher SNR and higher spectral resolution of TES as compared to CrIS. The retrieval up to 25 km for TP1 can be reliable for any shape of the IG. The retrievals of TP2 and TIP3 (Figure 2b) from IG1 are remarkably good. Tire above mentioned weak apace at ~ 10 km is also observed for TES solution and we need further studies of window/channel selection to improve the information at ~10 km by additional measurements. The RMSE value of surface temperature reduces further. The most interesting observation from this study is that the solutions of three Monte-Carlo simulations are almost identical as opposed to the same for CrIS. Three different noises in measurements produced three different retrieved profile shapes for CrIS. Although the solutions are convincing (especially for TES) as compared other published results, the exact solution is not achievable using these experimental setup (selection of channels). Thus, the focus of this study will be how much information can be extracted from the measurements by reduction of the IG error.

### 3.3. Information Content Analysis Using Subspace

Information content analysis of retrieval is a relative measure of the quality of retrieval for an applied method in some degrees. There is no absolute meaning of information content for parameter estimation from a remote sensing measurement and the estimated values are dependent on both forward and inverse modeling. For example, the number of degrees of freedom for a profile is effectively infinite (approximately the number of molecules along the path in the atmosphere) for RT modelling. It is not feasible to model this in a realistic situation due to the constraints in numerical modelling. Thus, in practice, a forward model is conventionally made with a finite number of grids (e.g., in this study, less than 1 km up to tropopause and then more than 1 km is considered). Such approaches restrict the degrees of freedom of the entire modelling scheme and the maximum number of pieces of information is restricted by the number of the state space parameters or the number of the measurements, whichever is lesser. One of the approaches for measuring the information content of stochastic-based retrieval is the degree of freedom from signal (DFS) [41], which is the trace of the averaging kernel (*A*) matrix of retrieval method. Analogous to *A* in the stochastic approach, model resolution matrix (*M*_rm_) is used in the deterministic approach [42]. The trace of the *M*_rm_ matrix is the degree of freedom in retrieval (DFR) (in the deterministic approach [43]). The total information content in terms of DFR can be calculated using the parameters of RTLS methodology at the final iteration as:

(7) $${\text{H}}_{\text{dfr}}=\mathrm{trace}\left({\left(K^{T}K-g(x)L+\alpha L^{T}L\right)}^{-1}K^{T}K\right)$$

**Figure 3.**
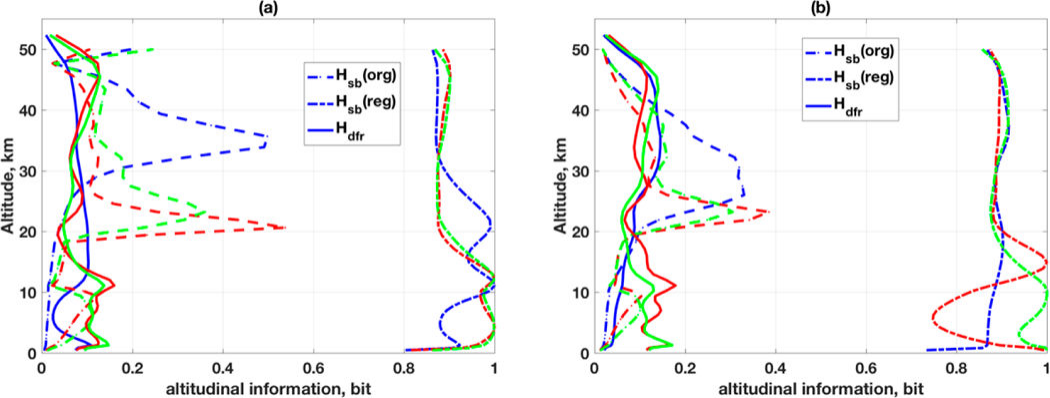
2D distribution of information content for simulated retrievals for three different profile shapes: TP1 (blue), TP2 (red) and TP3 (green) for (**a**) CrIS and (**b**) TES. Dashed lines: H_sb_ original H_sb_(org); Dashed-dotted lines: H_sb_ regularized H_sb_(reg).

Although H_dfr_ is a measure for understanding of tine quality of retrieval, it is unable to give a complete picture of how information is distributed along the attitude grid at every iteration. An assumption was made that the diagonal elements of *M*_rm_ is the information distribution along the altitudinal grids, which is plotted on Figure 3. The values of H_dfr_ are always less than 0.2 and well distributed . By definition the H_dfr_ of single altitudinal grid is 1for LS solution when measurement is more than or equal to the state space parameters. However, this reduces to ~0.2 (e.g., Figure 3) under ragularization using RTLS. One may argue that the solution contains 80% of noise if H_dfr_ is 0.2 but this ii not true in reality. Put differently, the value of H_dfr_ is not representative to understand how much error is propagated from the measurement space to the state space at the dime of inversion. Thus, we extend our study using subspace [44] analysis here. This idea is extended for vector space in such a way that the angle between two measurement vectors can represent the information added into the system by the second measurement with respect to the first measurement. One degree of freedom will be added if two measurement vectors are mutually perpendicular and an angle of 0 implies no information improvement by second measurement. It can be applied to state-space instead of measurement space, e.g., two state space parameters can be uniquely solved if two column vectors (a set of measurement) of the Jacobian, which are the derivatives of measurement/model with respect to the state space parameter, are perpendicular. [Please note that the variation of O_3_ number density can be viewed as a two-dimensional model, one within a given altitude and the other along the altitudinal path]. We calculate the rate of change of information along the altitude grids by calculating the angle between two adjacent column (measurements) vectors of the Jacobian as:

(8) $$\theta =\cos^{-1}\frac{K{\left(:,i\right)}^{T}K(:,j)}{\left\|K(:,i)\|\|K(:,j)\right\|}$$

where *i, j* are the adjacent column of the Jacobian. We have also verified our results using “subspace” routine available in MATL AB. The in formation is calculated as

(9) $${\text{H}}_{\text{sb}}=\sin\theta$$

We have calculated values of H_sb_ for the original Jacobian (***K***) for three different profiles as H_sb_(org) and inverted matrix of RTLS solution at the last iteration ([***K***; α***L***1; σ***L***1]) from IG1, which is the regularized Jacobian for the retrieved profiles as H_sb_(reg). The calculated subspace information far two different sensors are shown in Figure 3.

As mentioned earlier, a H_dfr_ value of 0.2 does not necessarily mean that there is 80% error. Take for example, in the case of CrIS (Figure 1), retrievals both for TP2 and TP3 are in weak space around ~10 km, but t lie H_dfr_ values are highest along the altitude and ~0.17. This cannot explain the true retrieval quality of an inversion. Contrary to H_dfr_-values, RTLS-based H_sb_(org) values (Figure 3a) clearly demonstrate lesser information contents along the altitude around ~10 km for both TP2 and TP3, which correspond to the weak-space retrievals as is seen in Figure 1. As a consequence of the weak space, RTLS inherently regularizes the original Jacobian ta a higher strength resulting in H_sb_(reg) value close to 1. This ensures a reduction in tide propagation of the random error, but the regularization error is relatively higher than other altitudinal grids and resulting; in d change in the shape of the retrieved profile. Although the values of H_sb_(org) are not: directly related to the quality o^f^ retrieval, the; consequences of the high values of H_sb_(or^g^) for TP2 and TP3 around ~20 km and for TP1 around ~35 km (Figure 3a) are not explored here. Similar weak space behavior is also noticed for TES simulated retrievals (Figures 2 and 3b) but in lesser magnitude due to the value of H_sb_(org) being higher than for CrIS and it is around ~15 km. Therefore, we would like to emphasize that only a straightforward averaging kernel or DFS/DFR analysis to understand the information content in the retrieved profiles is inadequate, as opposed to what has been a prevalent practice in the community.

Although the subspace information for two different sensors for same retrieval grids at original Jacobian space is significantly different, the distribution of altitudinal information H_sb_(reg) under RTLS regularization for both sensors are closer and near to 1. This confirms that the RTLS regularization scheme is optimally preventing the propagation of noise from the measurement space to the state space for almost all altitudinal grids and retrievals are reliable. The most striking result is that the full sinusoidal true profile shapes are closely retrieved (Figure 2) whereas the calculated DFR is only 3. Conventionally, a minimum of five pieces of information is required to resolve a full sinusoidal profile, which raises the question on the validity of the postulate of degree of freedom in retrieval or DFR. However, subspace information content analysis is also not fully conclusive, which we briefly discussed here. For example, the quality of retrieval is dependent on the noise in the measurements, which is not considered in the subspace information calculation and on the smoothing error due to different strengths of regularization. Although the smoothing error for both sensors may be the same due to identical regularization scheme, the higher error observed in CrIS is mainly due to the noise in measurements as we use simulated noisy spectrum for CrIS with SNR = 100 and for TES with SNR = 300. The number of selected channels for TES is eight times higher than that of CrIS, which also reduces the effective error in TES numerical experiment. The values of H_sb_(reg) close to 1 under regularization confirm that the error propagation from measurement space to state space is minimal. The ratio of the H_sb_ between the original and regularized Jacobian may be representative of the regularization error. These will be explored in a future study.

## 4. Simulated Profile Retrievals Using Radiosonde Data

**Figure 4.**
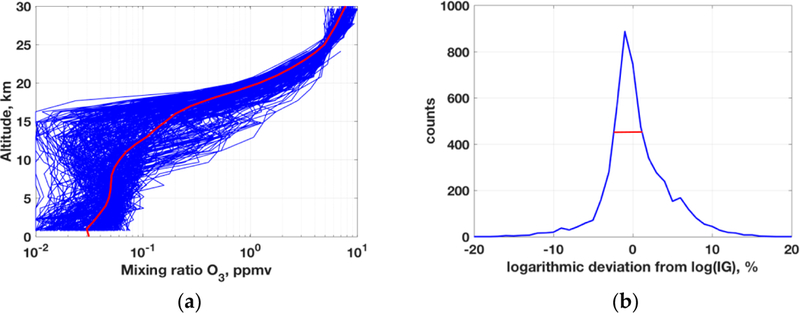
(**a**) A set of representative O_3_ profiles obtained from ozonesonde measurements. (**b**) Distribution of deviation from IG in percentage and logarithmic scale.

The OEM is one of the stochastic inverse methods gaining popularity in many remote sensing applications, since more than three decades ago [41] and maintaining it until recently [45]. However, some studies focused on the “deficiency” of OEM and employ additional constraints to the OEM method, e.g., by using Tikhonov regularization e.g., [46,47] or others. These enhancements are technically not parts of the original OEM approach but have been implemented to improve the OEM results. As such, there is nothing wrong in including additional constraints, but the issue is the lack of clarity in the retrieval, whether it is coming from adopted deterministic regularizations or from stochastic approximation processes inherent in OEM. Further to this, any occasional good result in operations may or may not be related to the core inverse method itself but is often attributed to OEM’s success. In contrast, a dynamic data-driven regularization is intrinsic to the RTLS method that also can be applied to a single measurement instance, unlike any stochastic approach, which by definition rely on assuming distributions of a prior, a priori error and measurement error. The iterative form of OEM [41] is:

(10) $$x_{\text{i}+1}=x_{\text{a}}+{\left(K^{T}S_{e}^{-1}K+S_{a}^{-1}\right)}^{-1}K^{T}S_{e}^{-1}\left(\left(y_{\delta }-y_{\text{i}}\right)+K\left(x_{\text{i}}-x_{\text{a}}\right)\right)$$

where, *x*_a_ is the a priori profile, *S*_a_ and *S*_e_ are the a priori and measurement error covariance matrices. These are additional parameters required only for OEM and a set of occurrences is required to develop a priori and measurement error covariance matrices. Thus, we conducted retrievals using a set of ozonesonde data collected from the Global Monitoring Division, Earth System Research Laboratory (ESRL) (ftp://aftp.cmdl.noaa.gov/data/ozwv/Ozonesonde/), representative of the earth’s atmosphere in conjunction with other collocated in situ parameters. We have collected 277 different in situ O_3_ profiles from this database (locations: Boulder, Colorado; Hilo, Hawaii; Huntsville, Alabama; Narragansett, Rhode Island; Pago Pago, American Samoa; South Pole, Antarctica and Suva, Fiji) to perform this simulation. The surface temperature (ST) was not available in this data base, thus adding 2 K to the near surface temperature data is set as true ST values. For simplicity, emissivity of the surface is assumed to be 1 for this simulation study, which is close to that of the ocean surface. The simulation has been made on the grid of the individual radiosonde profiles, thus, the different atmospheric grids are considered for different profile retrievals . The dataset has a mixture of different altitudinal coverage, e.g., some are up to 5 km while others Eire up to 30 km and the rest are in between. We have considered all the profiles to study extreme cases and construct a sound stochastic distribution. Although the major signal of measurement on O_3_ band is coming from the stratospheric level, the experiment has been done using only tropospheric O_3_ profiles up to 30 km because no in situ profiles data up to 50 km are readily available. This experiment can be viewed as a comparative lower-troposphere and upper-troposphere lower-stratosphere (UTLS) O_3_ retrieval using different inverse methods assuming that stratospheric O_3_ signal from satellite measurement can be successfully deducted. The plot of all profiles is shown in Figure 4 and an approximated middle profile (red) is considered to be a priori and IG for OEM and only IG for RTLS. Although IG is close to 1976 US standard O_3_ profile for the earth’s atmosphere, the variation of realistic O_3_ is huge (Figure 4), e.g., a range of ~3-ordtr of magnitude is observed at the tropopause (~15 Ion). This is a challenging problem to be solved using only 3–4 pieces of information from the measurements using any method. Some published papers reported that the success of OEM retrievals using a priori profiles is in getting results 1~2% close to true profiles e.g., [43,48]. However, the question remains whether it .a feasible to obtain such level of accurate a priori profile for satellite retrievals where the atmospheric variation is so large (Figure 4). In this experiment, the IG is set at 275 K for all surface temperature retrievals.

The information content associated with OEM retrievals in terms of the degree of freedom in signal (DFS) is given as:

(11) $${\text{H}}_{\text{dfs}}=\mathrm{trace}{\left(K^{T}S_{e}^{-1}K+S_{a}^{-1}\right)}^{-1}K^{T}S_{e}^{-1}K)$$

We calculated the stochastically exact a priori error covariance in terms of percentage of each individuil point from the a priori (red line in figure 4a) as is shown in Figure 4b, which certainly is advantageous for OEM as compared to in an operational setting with unknown a priori error covariance. The calculated a-priori error variance in terms of full width half maximum is ~6% for this dataset. Please note that the measurements are made by adding the random noise according to the SNR of the instruments on top of the calculated radiances using same ozonesonde profile data and as described earlier in Section 3.

### 4.1. Profile Retrievals Using RTLS

**Figure 5.**
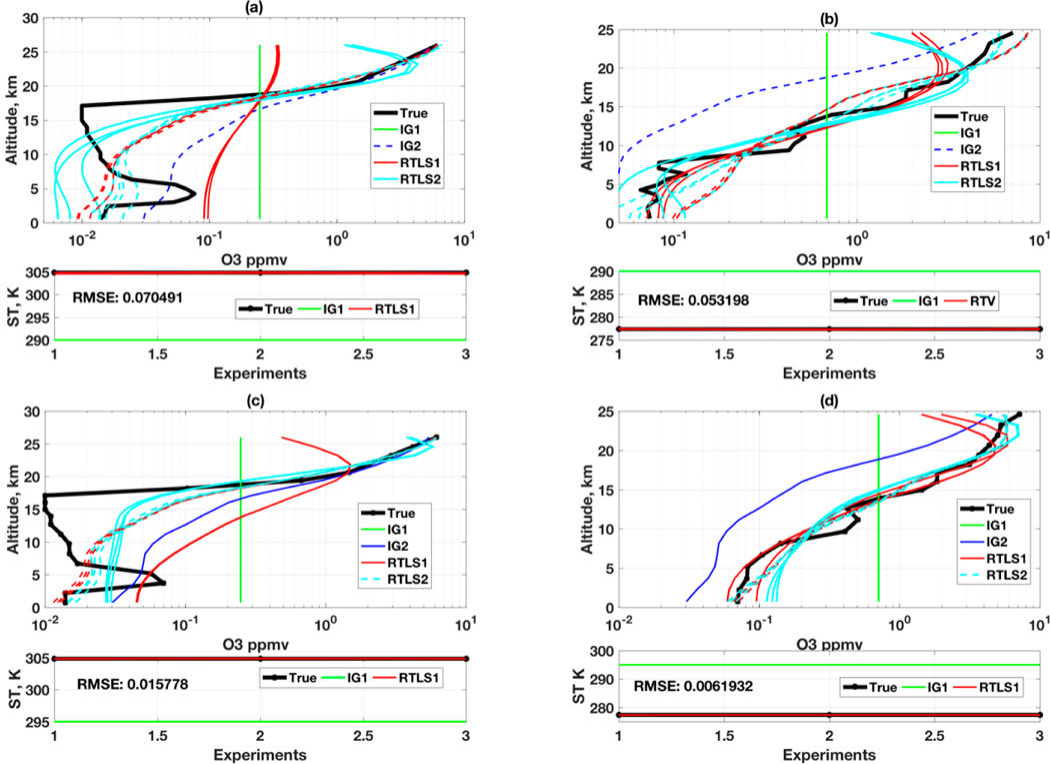
Retrievals employing both LFDO and LSDO in RTLS for two different true profiles and from two different IGs. Upper-panels (**a,b**): CrIS; Lower-panels (**c,d**): TE S. Colors: true profiles in black; two IGs in solid green and dashed blue; retrievals using LFDO in red and LSDO in cyan.

First, two distinctly different profiles are considered from this database to understand the different aspects of retrieval from realistic atmospheric measurements (ozonesonde). One of them is an extreme profile and the other one is comparatively simpler or close to straight-line profile. Although RTLS, usually uses a first derivative LFDO, it is intrinsically capable of also using a Laplacian second derivative operator (LSDO). In Figures 1 and 2, TP1 has a sharp peak and LSDO is not applicable (by definition it must fit a line through three consecutive updates and the presence of a sharp peak violates this requirement). However, in our experiment involving realistic radiosonde data (Figure 4a), there are no extreme peak as was shown in simulated profile in Figure 1. Therefore, we consider it worthwhile to test both LFDO and LSDO for improved understanding of the regularization process. The retrievals from two different IGs, two different derivative operators and two different instruments using RTLS are shown in Figure 5a–d (Figure 5a,b are for CrIS and Figure 5c,d are for TES). The two different IGs are chosen to represent a worst-case (vertical straight line in solid green) and a reasonable IG (dash blue line) as we defined earlier that is shown in Figure 4a. The retrievals employing LFDO (RTLS1) and LSDO (RTLS2) are shown in red and cyan colors, respectively. A retrieval shown in solid line corresponds to the use of the worst-case IG and a retrieval in dashed line is from the reasonable IG.

From Figure 5a,b, one can notice that the solution from extreme IG1 (solid green) improves using LSDO (solid cyan), as compared to LFDO (solid red). However, for a reasonable IG2 (dashed blue), the improvement is not noticeable. The failure of the LFDO solution from IG1 is following reason. LFDO solution unable to reconstruct the shape of the true profile when IG is far from truth (~2400%) because RTLS using LFDO is comparatively high regularize solution. Now coming to the true profile shapes, to resolve the extreme profile (Figure 5a) more than at least eight pieces of information are required, whereas regularized retrieval can provide maximum 2 to 3 pieces of information. Therefore, efficient regularization scheme smooths the solution adjusting The information content of the retrieval. Put another way, the measurement has all the information without regularization, but there is no solution without regularization due to the constraints of ill-conditioned inversions as discussed in our earlier publications [13–15]. The advantage of using RTLS is that it inherently determines the regularization strength compromising the information content of the retrieval and no noticeable oscillations observed in the solution around the inflection points. On the other hand, reasonable solutions are obtained from two different IGs using both LFDO and LSDO for a true profile devoid of major inflections (Figure 5b). Analogous analyses for TES are shown in Figure 5c,d and the results are very similar to those of CrIS, with an expected higher accuracy. The improvement of TES results for extreme true profile from both IGs is significant as shown in Figure 5c.

### 4.2. Profile Retrievals Using OEM

**Figure 6.**
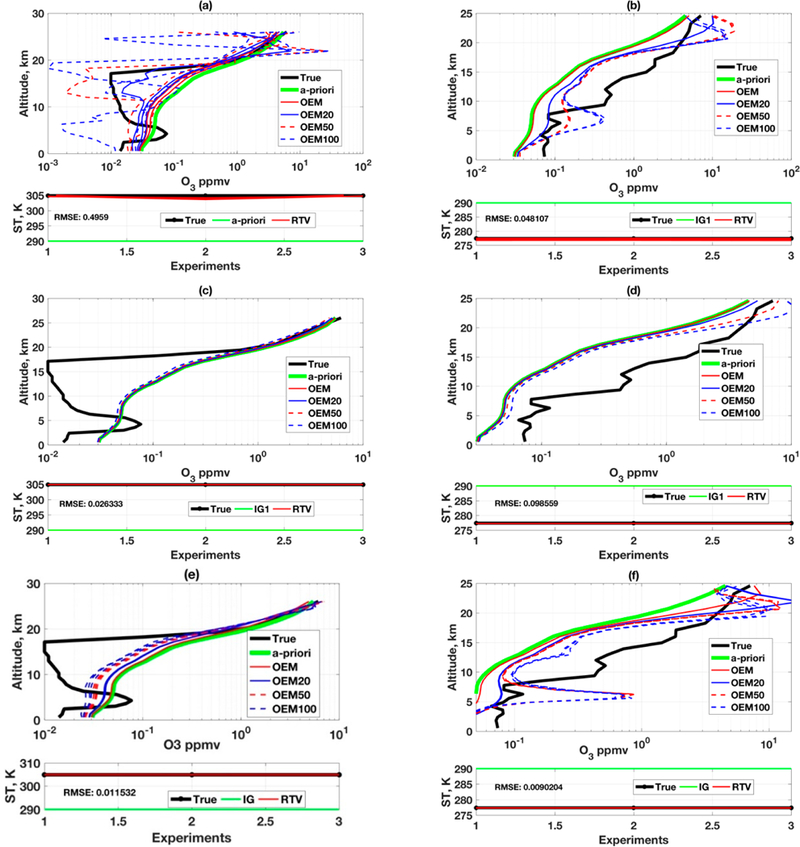
Retrievals in OEM for two different true profiles from a reasonable a priori. Upper-panels (**a,b**): CrIS; Middle-panels (**c,d**): TES with exact measurement error covariance (S_**e**_) at the rate of SNR = 300. Bottom-panels (**e,f**): same as in (**c,d**) but (DEM input with a higher noise level of SNR = 30. Color code: true; profiles in black; a priori in solid green; OEM (exact S_**a**_ and S_**e**_) in solid red; OEM20, OEM50 and OEM100 with tweaked S_**a**_ by increase of 20%, 50% and 100% in solid blue, dashed red and dashed blue, respectively. Left-panels: slightly complex true profile; Right-panels: comparatively simpler true profile shape.

In Figure 6a,b, it is observed that when so-called exact S_*a*_ and S_*e*_ are applied, OEM solutions (solid red) for CrIS simulations are close to the shape of the a priori implying a minimal extraction of information from measurements due to higher regularization in terms of regularization theory. The results of O_3_ retrieval are opposite to our previous observation on simulated H_2_O retrieval using of OEM [13], where solutions are highly oscillated under exact S_*a*_ and S_*e*_ due to very low regularization. This implies that OEM solutions perform inconsistently for different trace gas profile retrievals and for different case studies. This raises the fundamental question in applying OEM for satellite inverse problem when two similar profile retrieval problems will yield two different outputs when stochastically exact error covariances are used. This confirms that a method cannot reliably fulfill the scientific quest when the method is derived using fundamental flaw where error is treated as information.

Satellite retrieval community treats OEM as “accepted wisdom” and it is widely applied to many satellite retrieval problems without scientifically verifying the outcome of this method, which may result in devastating consequences on science developments. This, to our understanding, knowingly or unknowingly motivates researchers to apply tweaking to S_*a*_ and S_*e*_, perhaps without examining the root cause e.g., [4,45] to obtain some convincing output applying OEM. For example, there is no explanation for considering the 100% a priori error covariance in [45] and S_*e*_ is not estimated using the error distribution of forward model and instrument, but it is estimated from the fit residual [4]. Such approaches yield seemingly better results but are not mathematically or physically justifiable and high chances for information loss. To test such tweaking, we explored more simulations with increased of 20%, 50% and 100% S_*a*_ are shown in the same figure. This progressively yields profiles closer to the true profile, but still with significant oscillations. As compared to RTLS solution, OEM yields an inferior solution (see Figures 5 and 6).

In Figure 6c,d, retrievals for TES are shown under identical setting as for CrIS (Figure 6a,b). However, interestingly, the O_3_ profile retrieval outcome is even worse than that for CrIS that has a lower SNR (higher noise). Essentially, the solution closely follows a priori and information from (simulated) measurement is almost unused even after tweaking the S_*a*_. Please note, however, that the ST retrieval has slightly improved for TES (bottom panel of Figure 6c,d) as compared to the same for CrIS. One may argue that this happens due to the functional property of the TES measurements that may have “multiple solutions”. However, this is misleading since such inconsistency is not observed in the case of RTLS solutions shown in Figure 5c,d. This, therefore, accurately captures the inconsistencies in performance in OEM method when different instruments are simulated. In practice, one may tend to assign this non-performance to real instruments and further tweak S_*e*_ and other associated errors, but we demonstrated the root cause is the selected inverse method and not a nominally performing instrument. This issue is discussed further in the following paragraph.

The value of S_*e*_ is not only dependent on the sensor error only, but it also depends on forward model and other associated realistic errors in operation. Therefore, S_*e*_ is further increased (compared to reported sensor SNR of a particular instrument), perhaps based on trial and error, and the solutions achieve some meaningful numbers. The fundamental question is how to estimate the forward model error because it is almost impossible to develop a perfect forward for any science problem. While, the presence of associated errors in operation is not dismissed, this is one of the known drawbacks of applying OEM in operation. In controlled experiments (simulations), the “associated errors” including forward model are absent by choice. However, to test the outcome of tweaking S_*e*_, the input measurement error covariance values for OEM have been increased by ten times (1000% of its original), shown in Figure 6e,f. It is observed that the solution is improved by increasing the values of S***e***, but still inferior to RTLS solution. The “increase of S_*e*_” in this experiment is objectively reasonable according to regularization theory, which affects the solution in a way that the regularization strength is now reduced further on top of the tweaking of S_*a*_, therefore, facilitating extraction of some information from the measurements. However, the major argument is that the “increase of S_*e*_” does not have any scientific basis from stochastic covariance theory at least in the context of simulations where additional errors are absent.

### 4.3. Comparative Retrievals for CrIS

The current mainstream approach for parameter estimation from space-based measurement is based on constraining by climatological data as a priori and some additional ad hoc procedures on top of OEM. The main concern is how reliable climatological daily/monthly/yearly averaged state space parameter for the actual state when satellite measurements are conducted in under a minute for a highly dynamic atmosphere. Moreover, several ad hoc methods including different operational constraints are available in literature and it is not always comprehensible or straightforward to account for such constraints in simulated retrieval. Thus, a comparative study between the original inverse methods of deterministic and stochastic (with some tweaking of errors covariance) is discussed here.

**Figure 7.**
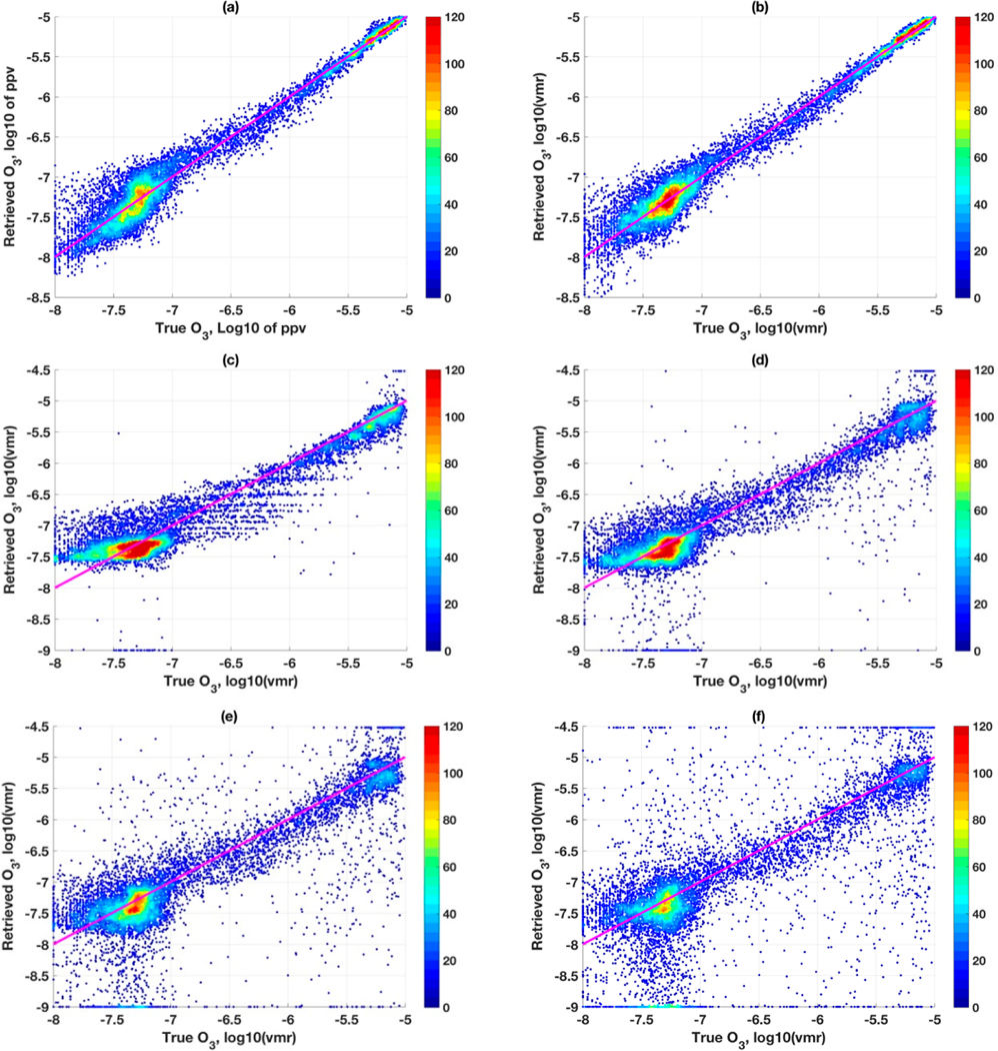
Bivariate density scatter plots between the individual points of simulated CrIS retrievals and 277 true ozonesonde profiles, (**a**) RTLSI employing LFDO; (**b**) RTLS2 employing LSDO; (**c–f**) OEM with four different a priori co-variances (6, 20, 50,100 percent, respectively).

RTLS solutions in Figure 5a,b can retrieve state parameters from CrIS simulated measurement unambiguously (with associated error and dependent on available information in measurement). These are significantly less error than those from OEM solutions in Figure 6a, under so-called exact error covariances. Recall that (Figure 5a,b, two profiles) LSDO performs slightly better than LFDO for most altitudes. For all 277 profiles (not two), we show results along the same line in Figure 7a,b but are presented in a different way that is convenient than line-plots to show multiple profiles. As is seen (Figure 7b), LSDO concentration follows a more systematic distribution, with high density at the core and less scatter confirming the observation seen from only two profiles (Figure 5) but for all profiles now. Please note that, since no error covariance is involved in RTLS, a single profile retrieval or collective retrieval of any number of profiles has no effect on the results. It can be concluded that the method is reliable and appropriate to be applied globally.

Figure 7c–f show OEM retrievals. Although error is known in this simulated study, to understand the effect of tweaking of a priori covariances on solutions, three additional irrationals a priori covariances of 20%, 50% and 100% are considered, which are shown in Figure 7d–f, respectively. The high systematic error observed in Figure 7c gradually decreases with increasing values of a priori covariances because of reducing regularization strength (in terms of deterministic interpretation). Please note that Figure 7c,f are the two extreme cases of S_*a*_, and Figure 7d,e are in between. While Figure 7c shows less scattered as well as less saturated (restrained boundary) points, Figure 7f shows significantly higher scatter and saturated points. However, the 1-to-1 ratio is stronger in Figure 7f as compared to Figure 7c. The peak-density reduces progressively between Figure 7c,f as those points contribute to the scatter, likely because of posterior error is higher than a priori error for high S_*a*_ values. Another interesting observation in Figure 7c is the presence of horizontal stripes. These stripes originate when posterior resembles the a priori, meaning that the algorithm did not yield any solution without further decreasing the regularization strength as shown in Figure 7d–f. It can be concluded from this study that optimum regularization for all iterations, by tweaking a priori error covariance, is almost impossible. The tweaking of error covariance or additional method on top of the OEM may improve the solution to some degree, but it poses more ambiguities and leaves us with several unanswered questions.

**Figure 8.**
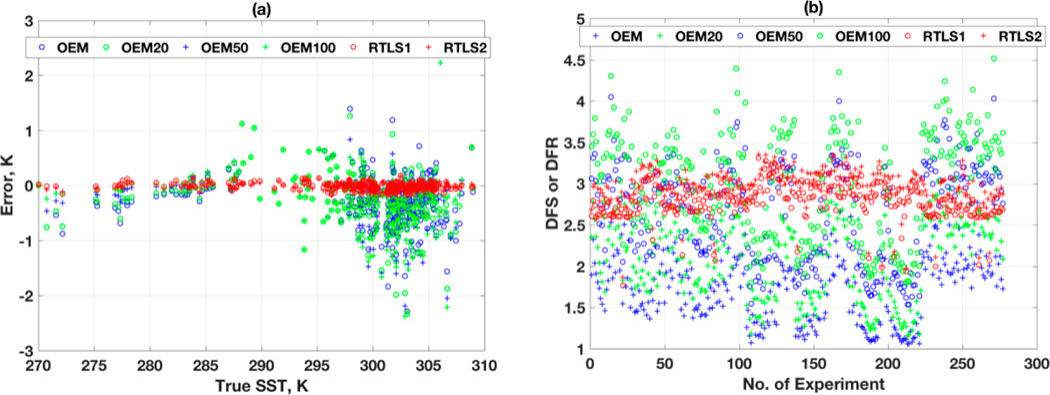
(**a**) Retrieval errors in surface temperature vs. true values, using RTLS and OEM; (**b**) Information content of the retrievals using; -various RTLS and OEM scheme.

Figure 8a shows the retrieval errors of ST using various schemes. The RMSE of retrieval using RTLS for all profile is 0.06 K, whereas the same for OEM with stochastically correct a priori error is 0.6 K. The ST error in OEM is one order magnitude higher than that of RTLS retrieval. The dispersion using OEM is more than ±3 K, whereas the same using RTLS is only ±0.2 K. Interesting results are found when inaccurate a priori errors are used: RMSE values are 1, 0.7 and 0.95 kelvins for a priori errors of 20%, 50% and 100%, respectively. This is counter-intuitive and cannot be explained only by simple regularization strength. Figure 8b shows the results for information content in terms of DFR and DFS at the last iteration of retrievals. The DFS (blue plus in Figure 8b) for the OEM solution when stochastically exact a priori error is used for all profiles are ~50% lower than the DFR using RTLS. It is also found that the DFS are highly dependent on the shape of the profiles as seen from scatter in Figure 10b, which is not the case for RTLS solution where DFR are consistent and close to 3. Out of three, one piece of information is observed for surface temperature and the other two are used for O_3_ profile retrieval. It is worth mentioning that an exact solution is impossible using only these two pieces of information for a profile with more than 10–15 unknowns. Therefore, inevitably there will be some error in retrievals regardless of any method employed and it is required to find a balance between reduction of error in state space arid maximizing the information extraction from the measurements. On the contrary, for OEM, the values of DFS are increasing with decreasing regularization strength (i.e., increasing a priori error covariances) and can reach values greater than 5 when large a priori error of 100°% is used. The high DFS solution can extract information from weak space of solution, but solutions are degraded by large random errors due to low regularization as show n in Figure 7f. For comparison, getting back to Figure 7a,b, RTLS regularizes the problem optimally and weak space information is smoothed using LFDO and LSDO.

### 4.4. Comparative Retrievals Results for TES

**Figure 9.**
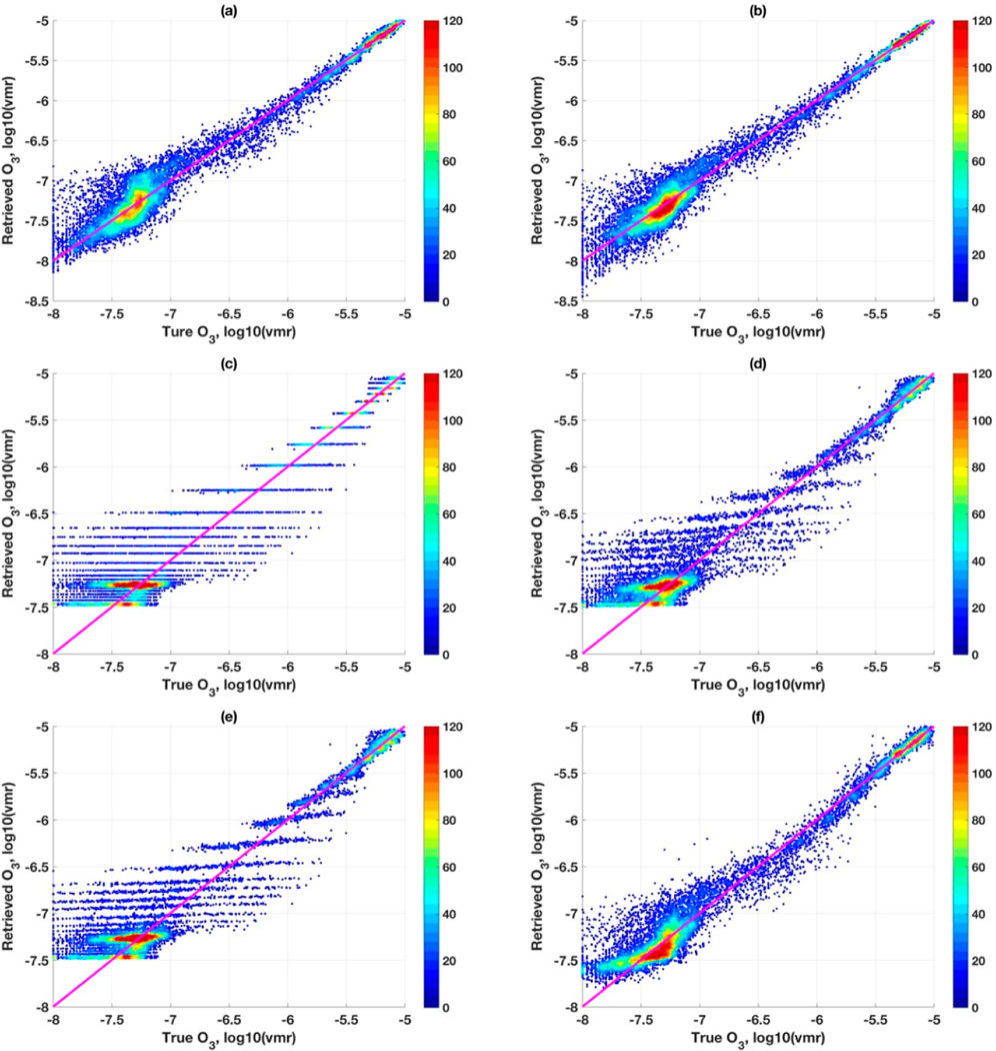
Bivariate density scatter plots between the individual points of simulated TES retrievals and 277 true ozonesonde profiles. (**a,b**) similar to Figure 7a,b; (**c–f**) OEM with two different a priori (S_*a*_) and two different measurement error covariances (S_*e*_). (**c**) S_*a*_ = exact, S_*e*_ = exact; (**d**) S_*a*_ = 100%, S_*e*_ = exact; (**e**) **S**_*a*_ = exact, S_*e*_ = 1000%> of original value; (**c**) S_*a*_ = 100%>, S_*e*_ = 1000% of original value.

The plots in Figure 9a–f are similar to as in Figure 7a–f but different settings were applied for OEM retrievals as OEM performed inconsistently between different sensors (CrIS and TES, here) as we have discussed earlier. Although the solutions of RTLS for TES in Figure 9a,b are approximately similar to the previous example of CrIS simulated retrievals in Figure 7a,b, the OEM solutions are far off from the previous case study. Figure 9c for OEM with “S_*a*_ = exact, S_*e*_ = exact” indicates that the solutions essentially fallback to the a priori values. Performances in Figure 9d,e with arbitrarily tweaking both the error covariances while holding one at its original value shows that the results are comparable (and both are unsatisfactory) with slightly more bias in Figure 9e. Interestingly, however, by extreme tweaking of both S_*a*_ and S_*e*_ as shown in Figure 9f, the solutions are reasonably good.

Recall that this last setting of CrIS (Figure 7f) which yields reasonable results, the settings for Figures 7f and 9f to achieve reasonable result using OEM are different. This confirms the performance of OEM are inconsistent for different characteristics of sensors. It is often reported in satellite retrieval literature e.g., [5–7] that there are inconsistencies in retrieved datasets from different sensors for a given trace gas. It is often argued that the measurement characteristics of different instruments are the primary cause for that. However, this study confirms that different tweakings of OEM are the root cause for final results. Moreover, using another experiment for the very selective dataset by fine tweaking of error covariances (chance success), one may attempt to show that OEM is a better inverse method compared to RTLS for a particular case study. However, fundamental assumptions to derive any stochastic inverse method are based on a random process, which prevents in investigating and understanding the cause and effect. This hinders further science development. For example, the residual analysis of a deterministic inverse provides an excellent opportunity to further improve the forward model whereas a stochastic inverse conceals these residuals inside the error covariances and bias correction.

**Figure 10.**
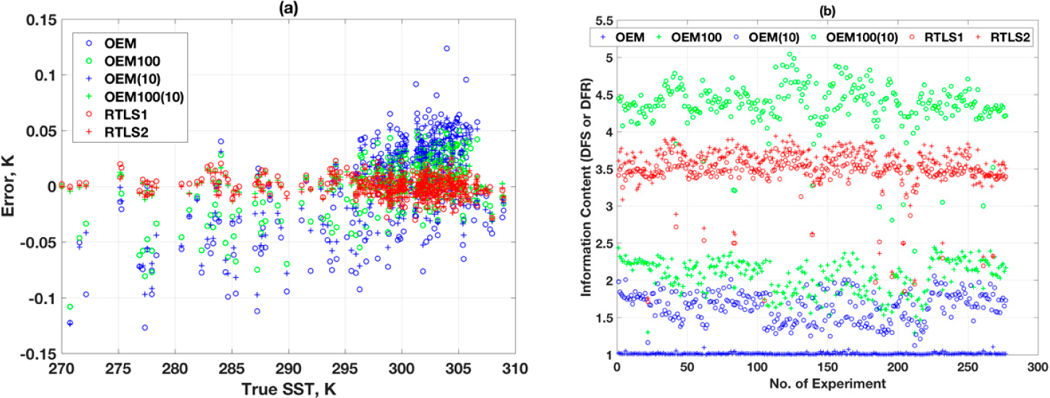
(**a**) Shows the retrieval errors oi ST using various retrieval schemes. The RMSE of retrieval using RTLS for all profiles is 0.007 K, whereas the same using OEM with stochastically correct a priori error is 0.05 K. The OEM error is one order magnitude higher than that using; RTLS. The dispersion of ST retrieval using; OEM is more than ±2 K, and for RTLS it is only ±0.2 K. (**b**) shows that the information content for LFDO (RTLS1) and LSDO (RTLS2).

Figure 10 is similar as Figure 8 but for the TES instrument. The RMSE of retrieval using RTLS (Figure 10a) for all profiles is 0.007 K, which is one order magnitude lesser than that for CrIS. The information content for RTLS is slightly improved for TES (Figure 10b) as compared to CrIS (Figure 8b), which is expected because SNR and spectral resolution of TES are higher than those of CrIS. For different OEM settings, the one corresponding to Figure 9d (i.e., S_*a*_ = 100%, S_*e*_ = exact) has slightly more information than Figure 9e (i.e., S_*a*_ = exact, S_*e*_ = 1000%). Repeatedly, this tweaking-related improvement does not warrant any scientific discussions as the reason is unclear. Tweaking both covariance errors surprisingly yields better solutions, but again the reason for it has no scientific basis.

### 4.5. Comparative Error Analysis between Both Sensors and Methods

**Figure 11.**
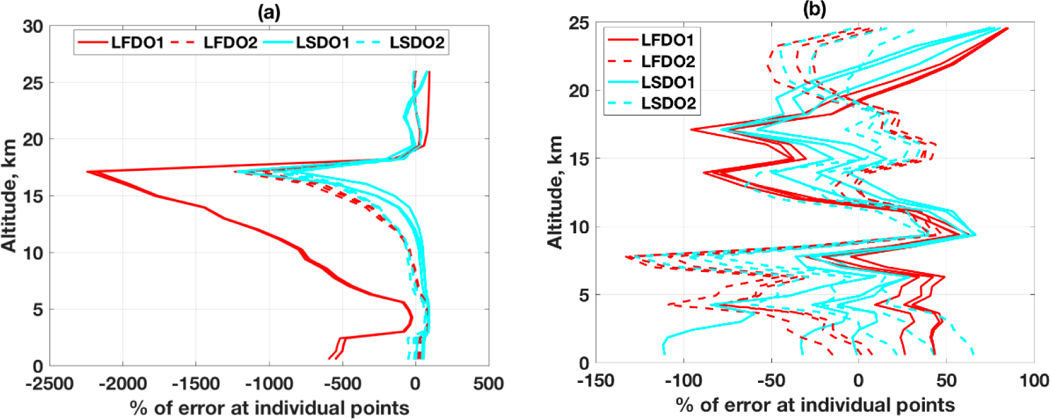
CrIS retrieval error on individual points of two different profiles (**a,b**) employing two different IGs as shown in Figure 7a,b (ignored here for brevity) and two different regularizations (LFDO, red; LSDO, cyan). The solution from two IGs are separated by solid and dashed data.

The error analysis of parameters’ estimation from satellite measurement is always a debatable issue due to lack of high-quality and abundant in situ measurements. Even it is sometimes argued that in situ measurement is also not “truth” due to different error patterns of different measurement systems. The only way a proper error analysis can be performed is through a controlled experiment with simulated retrieval where the truth is known, and the performance of the inverse methods can be well analyzed. We have already discussed rigorously in our earlier publication [13] that operational validation is made using tuned reference data sets with many ambiguous mathematical constraints and after discarding a significant number of retrievals for the sake of in situ quality control (e.g., Chi-squares test). A most debated current practice to validate the prevalent stochastic-based profile retrieval is that it needs alteration of the reference dataset with an averaging kernel of the inverse model e.g., [4,49–54]. Our aim is not to present a gimmick (e.g., “TES profiles agree within 5–10%, less than the variability in CO distributions obtained by both TES and the aircraft instruments [54]”) in peer-review literature. One can easily find in [54] that the a priori profile is inside of the posterior error bar, which implies that information is lost after using the measurement. In another published article, for validation purposes OMPS-LP measurements are required to be within 5° latitude and 10° longitude from the O_3_ station, and with in a 12 h time span around the sonde launch. For each sonde; profile, all collocated OMPS-LP observations are again averaged before the comparison [4]. In our opinion, such approaches using repeated aggregation is excessive and will smooth out much of the existing error distribution especially for chemically active gases, such as O_3_. We would rather present a comparative error analysis to evaluate the performance of inverse methods by considering simulated experiments for realistic situations (realistic O_3_ profiles) instead of fine-tuning; of end-numbers . Earlier the qualitative retrieval for realistic O_3_ profiles Eire shown in Figure 7a,b using RTLS and difficulties of the problem are discussed, but: when a numb is is assigned to this retrieval, the error can reach up to 2000% for realistic situation as shown in Figure 11.

As seen in Figure 11a and discussed earlier in Section 3, for a complex profile with multiple inflection points and high gradient, retrieval errors can be very high for some points. In such extreme cases, however, LSDO from a straight-line IG (IG1) still achieves remarkably less error than LFDO. This potential improvement will be explored in our future work to get the best results in extremely difficult cases. For a comparatively smoother tree profile (Figure 11b) point retrieval errors are within ~100% from any IG and both derivative operators. To conclude this particular discussion, we most exercise sufficient caution in reporting and interpreting errors, such as “5–10%” as these numbers are very subjective and depend on sob-selection and tweaking.

**Figure 12.**
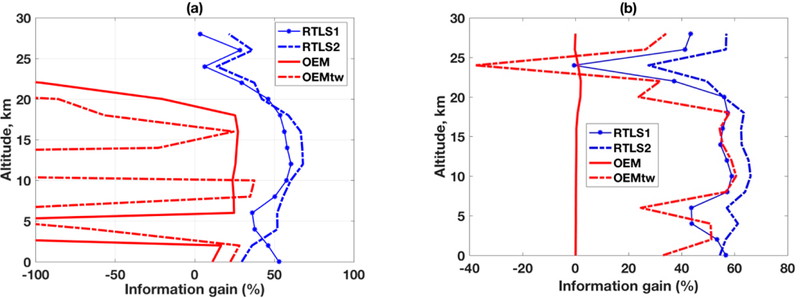
Information gain at different altitudes in comparison to percentage of IG error for different retrievals. (**a**) CrIS; (**b**) TES.

To give further comprehensive idea on the gross characteristics of the quality of the; retrievals for all profile!? employing different methods, we binned the data at 2 km interval, and calculated the root mean square error (RMSE) for each bin. The RMSE values have been calculated in terms of percentage of error as (δ*x*/*x*) for individual points as tire values of the O_3_ profile varies by more than two orders of magnitude along the profile that makes it difficult to show and interpret in its original value. Please note; that this inter-comparison study described below uses IG2 only (the straight-line IG is excluded) because OEM mechanism does not permit an unrealistic a priori and requires a close-to-truth profile as a priori. Also note that a priori and ICC are not necessarily the same, i.e., any a prior may serve as the IG, but the IG does not require a priori knowledge and it could be far from the truth or of a different shape from true profile. We have assumed that thee; IG and a priori are identical for this study. As mentioned earlier, an exact solution is not possible using a few pieces of information from measurements. The reconstruction of retrieved profiles using RTLS is dependent on the available DFR and the distance from the IG profile, and so is OEM by definition. Thus, the retrieval error is dependent on the IG error and we propose an innovative way to describe the inter-comparison study employing information gain (*G*_*inf*_) after measurement an the percentage of error reduction (PER) from IG error as shown in Figure 12. The G_inf_ is calculated as follows:

(12) $$G_{inf}=\epsilon_{ig}-\epsilon_{rtv;}\;\;where\;\varepsilon_{r}=rms\left\{\left(x_{r}-x_{true}\right)/x_{true}\right\}{\|}_{2km}$$

where, ϵ_*ig*_ and ϵ_*rtv*_ are the percentage of altitudinal RMSE for IG and re trievals from RTLS or OEM respectively. ε_*r*_ is the percentage of the altitudinal RMSE for the reference r, which can be IG, RTLS and OEM.

The simplest way to understand information gain from Figure 12 is by looking at the values to the left and right of the zero-point on the abscissa. Any point with less than zero indicates information loss (when compared to IG error) and vice verse. The legends for RTLS1 (LFDO), RTLS2 (LSDO) and OEM (S_*a*_ = exact, S_*e*_ = exact) have been already mentioned and are the same for two figures. Only OEM_tw_ is different between Figure 12a,b, corresponding to Figures 7d and 9f, respectively. Foe RTLS, the performances are satisfactory and similar for both the instruments, with LSDO overly outperform LFDO and there is no information loss (retrieval error is always lesser than IG error). The information gain above ~20 km is lower than that of the bottom part This is because IG error is already low above ~20 km (cf., Figure 4a) and there is little scope for gaining further information. On the contrary, OEM results drastically vary between the two sensors. In the case of CrIS in Figure 12a the loss of information is very high, and we have restricted the abscissa to ±100% for retaining the clarity of presentation. Generally, for CrIS (Figure 12a), it is observed that there is virtually no information gain using OEM (solid red line) and the results worsen with tweaking (dashed red line). The posterior error higher than a priori error using OEM is not reporting here the first time, it is often reported in published literature e.g., [9,48,54]. On the other hand, for TES in Figure 12b, there is no update of solutions using OEM and the results are very good after tweaking and is comparable to that of LFDO. It is possible to cherry-pick such random success or at times obtain good results for a given sensor by trial-and-error or tweaking, but this has no objective basis.

## 5. Conclusions

The paper compares performances of commonly used stochastic inverse and deterministic regularized total least squares (RTLS) methods for simultaneous retrievals of O_3_ profile and surface temperature using simulated data. To gain confidence in our approach, ozonesonde data were used to represent highly dynamic and realistic atmospheric states. We reaffirm that RTLS is one of the most effective inverse methods applicable for highly non-linear satellite inverse problems in conjunction with our earlier publications. Interestingly, OEM solution is highly regularized for O_3_ retrievals when exact error covariances are used, which is contradictory to our previous study on H_2_O profile retrievals where the problem was very lightly regularized for the same setup. The paper also clearly demonstrates that OEM produces contradictory results across different sensors and various tweaking conditions. This study, based on consistent RTLS solutions and inconsistent OEM solutions between two sensors, confirms that the prevalent inverse method in operation is the primary cause for inconsistent retrievals for same gas profile from different sensors.

RTLS performances are compared using two different stabilizers, namely LFDO and LSDO and LSDO is outperform than that of LFDO. RTLS retrievals are characterized using subspace analysis. It is found that inherent regularization scheme of RTLS can prevent noise propagation from measurement space to state space holding the information content more than 0.8 along the altitudinal grids. One of the major findings is that RTLS can extract information from the measurement optimally and ~50% “information gain” is possible from tropospheric O_3_ retrieval from CrIS or TES measurement. On the contrary, OEM often yields more errors than are present in the a priori, which leads to loss of information. In this era of advanced hyperspectral measurements from satellites, we emphasize that RTLS-based methods are capable of unambiguously converting “data to information” and should be further explored to improve present day retrievals.

## Appendix A

**Additional acronyms**
ACE-FTS: Atmospheric Chemistry Experiment-Fourier Transform Spectrometer
AIRS: Atmospheric InfraRed Sounder
GOME: Global Ozone Monitoring Experiment
IASI: Infrared Atmospheric Sounding Interferometer
MIPAS: Michelson Interferometer for Passive Atmospheric Sounding
MLS: Microwave Limb Sounder
OMI: Ozone Monitoring Instrument
OMPS: Ozone Mapping Profiler Suite
TOMS: Total Ozone Mapping Spectrometer
SBUV: Solar Backscatter Ultraviolet Radiometer
SCIAMACHY: SCanning Imaging Absorption SpectroMeter for Atmospheric CHartographY
